# Generalizations of Markov model to characterize biological sequences

**DOI:** 10.1186/1471-2105-6-219

**Published:** 2005-09-06

**Authors:** Junwen Wang, Sridhar Hannenhalli

**Affiliations:** 1 Penn Center for Bioinformatics, Department of Genetics, University of Pennsylvania Philadelphia, PA 19104-6021, USA

## Abstract

**Background:**

The currently used *k*^*th *^order Markov models estimate the probability of generating a *single *nucleotide conditional upon the immediately preceding (*gap *= 0) *k *units. However, this neither takes into account the joint dependency of *multiple *neighboring nucleotides, nor does it consider the long range dependency with *gap*>0.

**Result:**

We describe a configurable tool to explore generalizations of the standard Markov model. We evaluated whether the sequence classification accuracy can be improved by using an alternative set of model parameters. The evaluation was done on four classes of biological sequences – CpG-poor promoters, all promoters, exons and nucleosome positioning sequences. Using di- and tri-nucleotide as the model unit significantly improved the sequence classification accuracy relative to the standard single nucleotide model. In the case of nucleosome positioning sequences, optimal accuracy was achieved at a *gap *length of 4. Furthermore in the plot of classification accuracy versus the gap, a periodicity of 10–11 bps was observed which might indicate structural preferences in the nucleosome positioning sequence. The tool is implemented in Java and is available for download at .

**Conclusion:**

Markov modeling is an important component of many sequence analysis tools. We have extended the standard Markov model to incorporate joint and long range dependencies between the sequence elements. The proposed generalizations of the Markov model are likely to improve the overall accuracy of sequence analysis tools.

## Background

Biological complexity has evolved through a combination and interactions between simpler units. By looking at these units in a context dependent way, we can better understand the biological complexity. For example, Wang and Feng explored the amino acid propensity pattern in a neighbor-dependent way and found that the patterns were not always predictable from the single amino acid patterns [1]. Application of these di-amino acid propensity patterns into a traditional Needleman-Wunsch [2] algorithm significantly improved protein sequence alignment [3]. Similarly one can better predict the transcription factor binding sites by considering the interdependence between nucleotides [4,5].

Markov model (MM) is a statistical technique to model sequences such that the probability of a sequence element is based on a limited context preceding the element [6,7]. In other words, MM is a way to factorize the probability of observing the sequence in terms of context-dependent probabilities of the sequence elements. It has been effectively used in many DNA sequence recognition problems such as promoter and gene prediction [8]. The standard *k*^th ^order MM assumes that a sequence element probability depends on *k *previous bases, immediately preceding the current base. Alternatively, the standard Markov Model *generates *a single base (*model unit size *= 1) according to a probability distribution depending on the *k *bases immediately preceding the generated base (*gap *= 0).

The biological rationale behind selecting these parameters is not clear and alternatives should be explored. Longer range dependencies and joint dependency of neighboring bases have been observed in protein and DNA sequences. For instance, CG di-nucleotide is what characterizes CpG islands [1,9]. In bacterial promoters, a regular positioning of TA and TG stacks is prevalent with the best fit period 5.6 bp [10]. Stacking between neighboring bases is an important source of enthalpy change on helix formation [11]. In the study by Ozoline et al. the period of 5.6 bps for TA and TG can be interpreted as half of the helical repeat period with a contribution from a sequence-dependent helical writhe of the promoter DNA [10]. A repetition of certain di-nucleotide at 10–11 bp has been discovered in numerous genomes, supporting the DNA wrapping around the nucleosomes [12]. A model with unit size of 2 might be more appropriate to characterize the joint dependency of CG di-nucleotide. Furthermore, longer range dependencies (*gap *> 0) should be explored to model the periodicity of helix pattern. These alternative hypotheses regarding the positional and joint dependence within sequences can be computationally evaluated by extending the Markov Models.

There have been attempts to generalize Markov models. The *Mixture Transition Distribution Model *conditions the current state on a combination of previous states at varying distances [13]. In the *spatial model*, the current nucleotide depends on both the left and the right nucleotides [14]. For a detailed review of other generalizations and their limitations see [15]. We have developed a configurable tool to allow for generalizations of Markov model (GMM), as described in the implementation section.

We have evaluated a few instances of our GMM for their ability to classify four classes of sequences – CpG-poor promoters, all promoters, exons and nucleosome positioning sequences against appropriate background sequences. We compared two special cases of our model, the third order di-nucleotide (model unit size = 2) and 2^nd ^order tri-nucleotide (model unit size = 3) GMM against the traditional 6^th ^order single nucleotide Markov model. Our results based on 10-fold cross validation show that the di-nucleotide and the tri-nucleotide based models are significantly better than the single nucleotide based models. Furthermore, in the case of nucleosome positioning sequences, a *gap *length of 4 achieves the optimal classification accuracy. By allowing us to explore the dependence structure, the GMM tool will not only improve the classification accuracy of a sequence class, but will also provide insights into the structural properties of the sequences.

## Implementation

**Figure 1:**
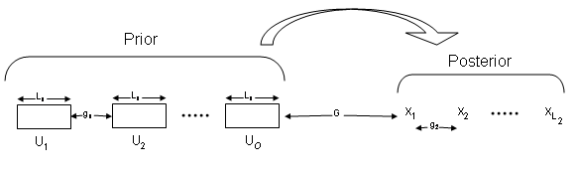
The figure illustrates the six configurable parameters. There are three parameter associated with the prior – model unit size *L*_*1*_, order (number of units) *0*, and spacing between units *g*_*1*_. There are two parameter associated with the posterior – model unit size *L*_*2*_, and spacing between bases *g*_*2*_. And there is a gap parameter *G*. Although not as general as arbitrary graphical models, this implementation is highly configurable with respect to model unit sizes and the dependence structures in terms of gap lengths.

We define the bases whose probability we wish to compute as the *posterior *bases or simply the *posterior *and the bases on which this probability is conditioned upon, as the *prior*. We use six parameters to specify a Markov model (as shown in Figure 1). To capture the joint dependency of neighboring nucleotides, our model allows multiple nucleotides as the model unit. However we allow different model unit sizes for the prior and the posterior, denoted as *L*_*1 *_and *L*_*2 *_respectively. The *gap *between the posterior and prior is denoted by *G*. The prior is composed of a few individual model units. The number of such units is called *order*. The maximum *order *is denoted by *O*. We also allow these individual prior units to be at an arbitrary spacing from each other. This spacing between the prior units is denoted by *g*_*1*_. Lastly, within the bases comprising the *posterior *we allow arbitrary spacing between the bases denoted as *g*_*2*_. For instance a spacing of length 2 within a posterior model unit of size 2 in an amino acid sequence captures the joint dependency for the first and the fourth amino acid residue, which is likely to form a hydrogen bond – vital for the protein helix structure [16]. To evaluate a model where each tri-nucleotide depends on the previous 4 bases, one can set *L*_*1 *_= 4, *0 *= *1*, *L*_*2 *_= 3, *g*_*1 *_= *g*_*2 *_= *G *= 0. To use the four bases after ignoring the immediately preceding 3 bases, one can set *G *= 3.

The prior order *O *only specifies the maximum order. Our program uses the idea of *variable length Markov model *[17] such that the highest order for which sufficient data is available, is utilized [18,19]. Apart from the 6 parameters mentioned above, the other generic configurable parameters include: type of biological sequence, either protein ('P') or DNA ('N'); threshold for minimal count of prior for *k*-mer elimination; pseudo count for a *k*-mer absent in the training set and the phase the user wants to score. For further information on the parameters, please refer to the software package readme file. Given a particular configuration, our implementation of the GMM is very similar to that of GLIMMER package, with a few exceptions.

### Training

In order to achieve statistical robustness, we only consider the *k*-mers above a (configurable) frequency threshold in the positive sequences. This frequency must ensure that the estimated conditional probabilities are acceptably close to true probabilities. A frequency threshold of 400 was estimated in [19] that provided a 95% confidence that the estimated probabilities were within 0.05 from the true ones. We tried varying this threshold from 50 to 500 and it did not make a significant difference in performance (also observed in [19]) and attains the maximum at 300. Hence we chose this as the default threshold. For nucleosome sequences we chose 50 as the frequency threshold due to smaller data set.

We slide the window one base at a time along the training sequence. The window size is determined by the user defined parameters; window size = *L*_*1 *_× *O *+ *g*_*1 *_× (*O*-*1*) + *G *+ *L*_*2 *_+ *g*_*2 *_× (*L*_*2*_-*1*). For each window, we extract the words corresponding to the prior and the posterior. For example, for *L*_*1 *_= 1, *O *= 6, *L*_*2 *_= 2, *g*_*1 *_= 0, *G *= 1, *g*_*2 *_= 1, we have a window with length 10, say ACTGATGCAG. The di-nucleotide CG represents the posterior. We increment the counts of k-mers ACTGATCG (6^th ^order), CTGATCG (5^th ^order), ..., and CG (0^th ^order) by one. We thus have 7 sub-models, one for each order.

Once the training sequences are processed, we convert the raw counts into transition probabilities. For the 0^th ^order, the probability is the composition of the *L*_*2*_-mers. For higher order, say, 4^th ^order TGATCG, we compute the sum of frequencies of all the hexamers of the form TGAT**. If the sum is bigger than the user specified threshold, we calculate the probability by dividing the count of TGATCG by the sum. Otherwise, the program automatically uses the (*k*-*1*)-mer, and so on to order 0, where the base composition is used. The same process is repeated for the background training sequences and we thus obtain a negative model. We then convert the probability for each *k*-mer into log-odds score.

### Testing

The program first reads in the model – the *k*-mer log-odds – along with the configuration file. Scoring proceeds in a sliding window fashion where each window is the minimal sequence containing a posterior and the prior as described above. To score a window, we first consider the highest order. Using the example above, to score ACTGATGCAG (the underscored bases correspond to gaps in the model), we first look for 6^th ^order dependence, ie., ACTGATCG in the 8-mer table. If the string exists, we use the score. Otherwise, we look for the string corresponding to the 5^th ^order (CTGATCG), and so on, until the 0^th ^order, ie., the di-nucleotide composition. The sequence score is obtained by adding all window scores. We score the sequence using two models corresponding to the positive and the negative sequences.

For posterior length *L*_*2*_, the overall sequence score can be interpreted as the sum of the scores of *L*_*2 *_independent *parses *of the sequence in different *phases*. In each parse or phase, any given base is generated exactly once. We will illustrate this with an example. Let *L*_*2 *_= 3, and *g*_*2 *_= 1. Consider a test sequence *S *= *s*_*1*_*s*_*2*_...*s*_*n*_. The posterior *P*_*i *_starting at i^th ^position is *s*_*i*_*s*_*i*+*2*_*s*_*i*+*4*_. Each *P*_*i *_is in a specific phase φ_k_, 1 ≤ κ ≤ *L*_*2*_. Under φ_1 _we consider *P*_*1*_,*P*_*2*_,*P*_*7*_,*P*_*8*_,*P*_*13*_,*P*_*14*_, .... We jump from *P*_*2*_* to P*_*7*_, since all bases from *s*_*1 *_to *s*_*6 *_are covered by *P*_*1 *_and *P*_*2*_. Similarly under φ_2 _we consider *P*_*3*_,*P*_*4*_,*P*_*9*_,*P*_*10*_,*P*_*15*_,*P*_*16*_, .... Hence the phases for *P*_*i*_, i = 1,2,3..... are 1,1,2,2,3,3,1,1,2,2,3,3.... Note that each base position is covered exactly once in any of the three phases. If one has a prior knowledge of sequence phase (eg. in-phase exons) and does not wish to use the sum of all phases as a sequence score, one can specify a particular phase to be used. The model will use only the posteriors in that specific phase for training and scoring.

## Results

### The human promoter sequences

We extracted the ± 5 kb region around the 12,333 Transcriptional Start Site (TSS) in the DBTSS database [20]. These start sites are identified using *oligo-capping *approach. We have implemented a sliding-window based program to identify CpG-islands using the original definition of CpG-islands [21]. We have also implemented a Hidden Markov Model (HMM) approach for CpG island identification [7]. We call a 10 kb promoter region CpG-poor if it does not contain a 200 bp length CpG-island by either of the two methods. This resulted in 1,466 CpG-poor promoters from a total of 12,333 promoter sequences. We then randomly selected 5,000 10 kb sequences from the whole human genome after masking the DBTSS promoter regions. The 5,000 background sequences and the 1,466 CpG-poor promoters were used to evaluate the various models. The same background dataset was also used for the classification of the entire set of 12,333 promoter sequences.

### The human exon dataset

The human exon locations were downloaded from UCSC genome browser, human genome version hg16. We extracted the exon sequences based on start and end locations. We thus obtained 219,624 exons. To compile a background sequence set, we randomly selected the same length sequences from the background for each exon.

### The nucleosome positioning sequences

The nucleosome positioning sequences were downloaded from the Nucleosome Positioning Region Database (NPRD) [22, 31]. The generation of background sequences was done similarly to the exon dataset.

### Model evaluation

We used 10-fold cross-validations to train and test the models. The positive and the background sequences were randomly partitioned into 10 equal parts. Each part was tested after training on the other 9 parts. Once the models were trained, we scored the training set using the models and obtained a cutoff based on the specificity-sensitivity curve. We chose a score cutoff that resulted in the best correlation coefficient (CC) value for the training set. We then scored the (independent) test set and applied this cutoff to obtain the CC value. The mean and standard deviation over the 10 CC values was calculated. The Sensitivity (S_e_), Specificity (S_p_) and Correlation coefficient (CC) values were defined as following:



*TP: *True positive, *FP: *False positive, *TN: *True negative, *FN: *False negative.

We have provided scripts to evaluate a specified configuration based on 10-fold cross validation. This involves scripts for splitting the input sequence into 10 equal parts and code for calculating the sensitivity, specificity and correlation coefficient.

To assess the significance of the performance improvement using a model M compared to base model M* (standard MM), we used Wilcoxon paired rank sum test. All sequences (positive and background) were scored using M to obtain score list S and using M* to obtain score list S*. Both S and S* were normalized separately to mean 0 and standard deviation 1. These paired normalized scores for positive sequences (each sequence has 2 scores corresponding to the 2 models) were used to test whether the scores in S* are greater than the corresponding scores in S using Wilcoxon test.

We have applied specific configurations of the tool to a few biological sequence classification problems as an illustration. Specifically to evaluate the impact of varying model unit size we used the following three settings:

(1) 6^th ^order single nucleotide model: ***L*_*1 *_= *L*_*2 *_= 1, *O *= 6, ***g*_*1 *_= 0, *G *= 0, *g*_*2 *_= 0,

(2) 3^rd ^order di-nucleotide model: ***L*_*1 *_= *L*_*2 *_= 2, *O *= 3, ***g*_*1 *_= 0, *G *= 0, *g*_*2 *_= 0,

(3) 2^th ^order tri-nucleotide model: ***L*_*1 *_= *L*_*2 *_= 3, *O *= 2, ***g*_*1 *_= 0, *G *= 0, *g*_*2 *_= 0.

The 6^th ^order single-nucleotide Markov Model is common in many sequence analysis tools currently used. Notice that the total number of *prior *bases is six for each of these three models. We tested the classification accuracy for three sequence classes using the above three configurations. The results for CpG-poor promotes, all promoters and all exon classifications are showed in Table 1, and discussed below.

**Table 1:** Average and standard deviation of Correlation coefficient (CC) values using different models. The data were obtained from 10 cross-validation. The CC values were obtained from testing dataset when cutoff selected from the training set. * Wilcox rank sum paired test shows significant (p-value < 0.001) better than the corresponding single nucleotide model.

**Samples (size)**	**Single nucleotide**	**Di-nucleotide**	**Tri-nucleotide**
**CpG-poor Promoters (1,466)**	0.24 ± 0.05	0.28 ± 0.03*	0.34 ± 0.04*
**All Promoters (12,333)**	0.54 ± 0.02	0.54 ± 0.03	0.56 ± 0.02*
**All Exons (219,624)**	0.63 ± 0.00	0.64 ± 0.00*	0.67 ± 0.00*

### Classification of CpG-poor promoters

**Figure 2:**
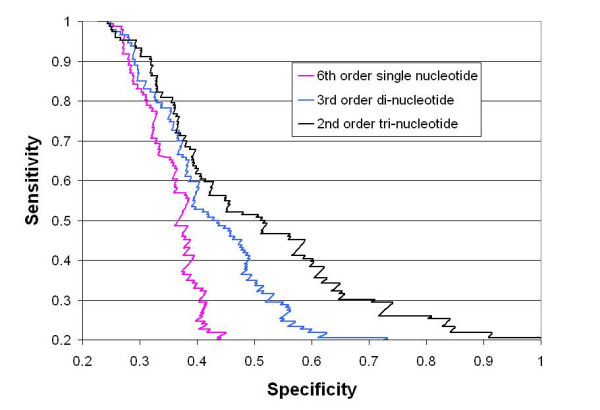
The specificity-sensitivity graph of the discrimination of CpG-poor promoters against background sequences using three different models – 6^th ^order single nucleotide model (Red), 3^rd ^order di-nucleotide model (Blue), and 2^nd ^order tri-nucleotide model (Black).

The di- and tri- nucleotide models improve upon single nucleotide model (p-value < 0.001). The traditional (single-nucleotide) 6^th ^order Markov model yielded a correlation coefficient value (CC) of 0.24. When we use the tri- nucleotide model, the CC value was improved by 39% to 0.34. The specificity-sensitivity graph (Figure 2) further shows the sensitivity-specificity tradeoff. For instance, at a reference sensitivity value of 0.5, the specificity achieved by the tri-nucleotide based model is 0.52, as compared to 0.36 for the single-nucleotide model.

### Classification of all promoters

We next applied the models to classify the entire set of 12,333 promoter sequences. The tri-nucleotide model shows an improvement in the classification accuracy (0.58 versus 0.54, p-value < 0.001) relative to the single nucleotide model. The entire set of promoters is dominated by CpG associated promoters which by virtue of being GC-rich and containing CpG islands have a strongly distinguishable characteristics against the background sequences. Consequently the relative gains of using larger model units are marginal.

### Classification of exons

We extracted 219,624 annotated exons from the hg16. We randomly selected the 219,624 sequences with the same length as the exons from background sequences. The average correlation coefficient for classification accuracy for single-, di-, and tri-nucleotide models are 0.63, 0.645 and 0.66 respectively. This modest improvement is however statistically significant.

### Classification of nucleosome positioning sequences

**Figure 3:**
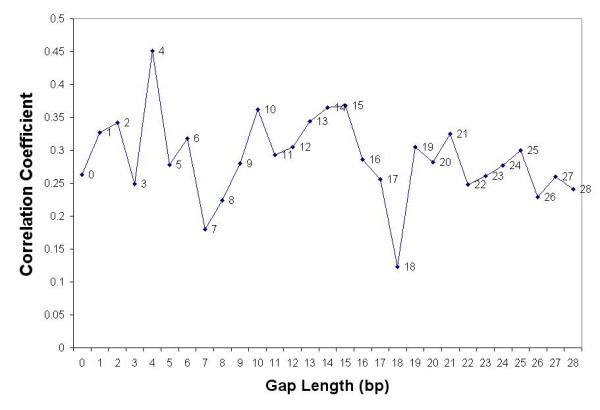
Plot of classification accuracy for the Nucleosome positioning sequences with respect to the gap between the prior and the posterior. This is based on the first order tri-nucleotide model (*L*_*1 *_= 3, *O *= 1, *g*_*1 *_= 0, *L*_*2 *_= 3, *g*_*2 *_= 0) at varying values of *G*. We achieve the best classification accuracies (peaks) at G = 4, 15 and 25, and worst classification accuracies (valleys) at G = 7 and 18. The distances between consecutive peaks and valleys are around 10–11 bps, which is close to DNA helix turn of 10.5 bps (for most common B-DNA, 11 bps for A-DNA, 12 bps for Z-DNA). This result illustrates the utility of the tool in exploring such long-range dependencies which might indicate specific structural constraints of the sequence class.

A periodical distribution pattern of transcription factor sites was observed in promoter region that suggested a correlation between the positioning of nucleosomes and transcription factor binding sites [23]. To investigate the nucleosome sequence periodicity, we compared classification accuracy at different gap length (parameter *G*) between prior and posterior. We were able to obtain 112 nucleosome sequences and performed their classification based on the first order tri-nucleotide model (*L*_*1 *_= 3, *O *= 1, *g*_*1 *_= 0, *L*_*2 *_= 3, *g*_*2 *_= 0) at varying values of *G*. We achieve the best classification accuracies at G = 4, 15 and 25, and worst classification accuracies at G = 7 and 18 (Figure 3). The distances between consecutive peaks and valleys are around 10–11 bps, which is close to DNA helix turn of 10.5 bps (for most common B-DNA, 11 bps for A-DNA, 12 bps for Z-DNA).

### Run time

We compared the run time for the three models on training and testing of the CpG-poor promoter classification against the background. The benchmark was based on 64 Mb sequences with parameters described in the method section. The java program was tested on a 2.6 GHz Pentium III dual processors with 16GB of RAM running linux. The training time for single-nucleotide based model was 55.8 minutes. This reduced to 23.8 and 18.9 minutes for the di- and tri-nucleotide based models respectively. The time needed for testing reduces less significantly by 30%-40%, from 22.9 minutes for single to 15.4 and 14.0 minutes for di- and tri nucleotide models respectively. The run time reductions are mainly due to fewer orders the model needs to go though for di- and tri- nucleotide models.

## Discussion

Markov chains are commonly used to model biological sequences. However the specific model unit size and the dependence structure among the sequence elements have not been explored. Specifically the model unit is fixed as a single nucleotide or amino acid and its dependence on *k *elements immediately preceding the current element is incorporated in the model. We have argued that it might be better to consider different model unit size and dependence structures. Furthermore, it has been reported that the optimal choice of model type and the model order is species specific [18]. Hence, it is important to implement the modeling tool in a configurable fashion.

### Promoter prediction

Despite numerous efforts in promoter prediction, the subclass of promoters not associated with CpG islands or CpG-poor promoters are notoriously difficult to characterize and predict. This remains the main bottleneck in overall promoter prediction accuracy and an accurate analysis of transcriptional regulation [24-26]. One component of promoter prediction is a better characterization of overall DNA structural feature in the vicinity of the promoters. Consistent with other studies that by considering the neighboring dependency of amino acids can improve the protein sequence alignment [3], here we show that by using the longer Markov unit to capture the joint dependency of neighboring nucleotides, we can substantially improve the CpG-poor promoter classification. Although we are not proposing an improved promoter prediction tool here, our result does suggests an alternative modeling of the long range DNA characteristics which is likely to improve the overall promoter prediction.

### Nucleosome positioning (NP) sequence prediction

The nucleosome is the basic unit of chromatin. Regulation of eukaryotic gene transcription is closely linked with the changes in nucleosome structure of the chromatin [22]. A nucleosome at the promoter region is capable of inhibiting the transcription initiation, whereas its displacement is capable of surmounting the repressive effect [27]. The preference of various sequences to allow for NP is not clear. An interesting aspect of our application of GMM to NP sequences is the observation that a gap length of 4 better captures the local dependence in these sequences. This, along with the periodicity of 10–11 bps in the plot of classification accuracy against the gap length might indicate a structural requirement in protein-DNA interaction in the NP.

### Generalizations of MM

Two main challenges in generalizing Markov models are (i) ensuring that the score of a sequence given the model can be appropriately factorized in terms of individual model unit scores (each base is included in exactly one model unit, modulo the edge effects), and (ii) accurate parameter estimation. We have shown that the sequence score can be interpreted as sum of scores using *L*_*2 *_independent parses of the sequence, where *L*_*2 *_is the number of posterior bases. Score of the sequence for each phase can indeed be factorized in terms of scores of disjoint posteriors. However with respect to accurate parameter estimation we have adopted a simple strategy analogous to that for standard MM and the parameter estimation methods developed in [15] may provide more accurate models.

We have used the *sum *of scores in different phases as the overall sequence score. Using the *maximum *score among all phases presents another alternative, which might be appropriate for coding exons where the codon impose a phase. When we do not have such *a priori *knowledge, then using maximum among phase scores may be inappropriate. Also it can be computationally prohibitive since one will need to build separate model of each phase and when scoring a sequence, try all models for all phase structure of the sequence. Thus the computational time for scoring a sequence is L_2 _*L_2 _– fold greater than the phase-less scoring. In our current implementation, for the cases where there is a prior knowledge of phase, users can specify a phase parameter, such that the model is built for a specific phase and also applied to the same phase.

## Conclusion

We have developed a configurable tool to explore generalizations of Markov models incorporating joint and long range dependencies of the sequence elements. As an illustration, we have shown that by using longer k-mer as Markov model units and specific gap lengths, one can improve the classification accuracy for a variety of biologically important sequence classes. Various tools to predict biological sequences like promoters and genes exploit multiple sequence based characteristics. The long range DNA characteristics are commonly captured using Markov models, eg Genscan [28] and HMMgene [29]. An improvement in this aspect of the prediction has direct implications on overall prediction accuracy of these tools. A complete theoretical development of generalizations of Markov models will require further research. The proposed software provides a means to explore dependency structures for a novel sequence class.

## Availability and requirements

The software will be freely available for download . The program requires java version 1.4.2 or above to run, and it is platform independent. Please refer to software package for detailed instruction on how to run the programs.

## List of abbreviations used

MM – Markov model

GMM – Generalizations of markov model

TSS – Transcription start site

DBTSS – Data base of transcription start sites

NP – Nucleosome positioning

NPRD – Nucleosome positioning region database

CC – Correlation coefficient

## Authors' contributions

JW and SH were involved in the developing the idea and writing the manuscript. JW implemented the software.
